# Recurrent Cholinergic Crisis Caused by Therapeutic-Dose Rivastigmine Patch in an Elderly Patient With Alzheimer's Disease: A Case Report

**DOI:** 10.7759/cureus.87868

**Published:** 2025-07-13

**Authors:** Kazuyoshi Matsuura, Taku Mayahara, Tomohiro Katayama, Hiroyuki Arai

**Affiliations:** 1 Department of Emergency and General Medicine, Kobe Ekisaikai Hospital, Kobe, JPN

**Keywords:** alzheimer's disease, cholinergic crisis, misdiagnosis, rivastigmine, therapeutic dose, transdermal patch

## Abstract

Cholinesterase inhibitors (ChEIs) are widely used for the treatment of dementia and other conditions, but may rarely cause cholinergic crisis, a potentially life-threatening complication. We report an elderly female patient with Alzheimer's disease who experienced three episodes of cholinergic crisis over 32 months while receiving a therapeutic dose of transdermal rivastigmine (18 mg/day). Each episode involved vomiting, diarrhea, diaphoresis, and neurological symptoms, with marked reductions in serum cholinesterase levels (53-63 U/L at presentation). During the first two episodes, alternative diagnoses such as acute gastroenteritis and possible pesticide exposure were initially suspected, and cholinergic crisis secondary to rivastigmine was not recognized. After the third episode, rivastigmine was permanently discontinued, resulting in the complete resolution of both acute and chronic mild gastrointestinal and autonomic symptoms. This case highlights the diagnostic challenges of cholinergic crisis in elderly patients receiving ChEIs and underscores the importance of considering this condition when unexplained gastrointestinal or autonomic symptoms occur even during standard therapeutic dosing.

## Introduction

Cholinesterase inhibitors (ChEIs) are widely prescribed for the treatment of Alzheimer's disease and other dementias. These agents enhance cholinergic neurotransmission by inhibiting the breakdown of acetylcholine, a chemical messenger critical for memory and muscle function, through the suppression of cholinesterase activity. Although ChEIs are generally well tolerated, they can, in rare instances, lead to cholinergic crisis, a potentially life-threatening condition caused by excessive acetylcholine activity resulting in the overstimulation of the parasympathetic nervous system. Clinical manifestations typically include gastrointestinal symptoms such as vomiting and diarrhea, autonomic symptoms like diaphoresis and miosis, and neuromuscular abnormalities such as muscle weakness or fasciculations. However, diagnosis can be challenging, particularly in elderly patients, where nonspecific presentations may mimic common medical conditions such as infections or intoxications.

In Japan, distigmine is the most frequently reported cause of ChEI-associated cholinergic crisis, particularly in patients treated for voiding dysfunction [1]. In contrast, rivastigmine, which is approved for the treatment of dementia and available in a transdermal patch formulation, has rarely been implicated. The transdermal route was specifically designed to provide more stable systemic absorption, thus reducing fluctuations in drug levels that might otherwise cause side effects, and to reduce gastrointestinal symptoms [2]. Nonetheless, there have been occasional reports of rivastigmine-induced cholinergic crisis, mostly in the setting of overdose [3-5]. Cases occurring under standard therapeutic dosing remain exceedingly rare, both in Japan and globally.

Here, we report a case of an elderly woman with Alzheimer's disease who experienced three separate episodes of cholinergic crisis while using a stable therapeutic dose of rivastigmine transdermal patches. Each episode was initially misdiagnosed as a more common illness, resulting in delayed recognition. This case highlights the diagnostic pitfalls and the importance of considering cholinergic toxicity even during routine ChEI therapy, especially when gastrointestinal or autonomic symptoms are unexplained. Early recognition of this rare but serious side effect may help avoid unnecessary investigations and enable timely, appropriate management.

## Case presentation

A woman in her 80s with Alzheimer's disease, hypothyroidism, chronic rhinitis, and a history of cholecystectomy was receiving rivastigmine at a dose of 18 mg daily, along with multiple concomitant medications including gastroprotective agents, antitussives, herbal medicines, and antihistamines. Over approximately 32 months, she experienced three separate episodes of acute illness requiring emergency transportation, each characterized by gastrointestinal and neurological symptoms. There was no evidence of rivastigmine overdose in any of the episodes.

First episode

**Figure 1:**
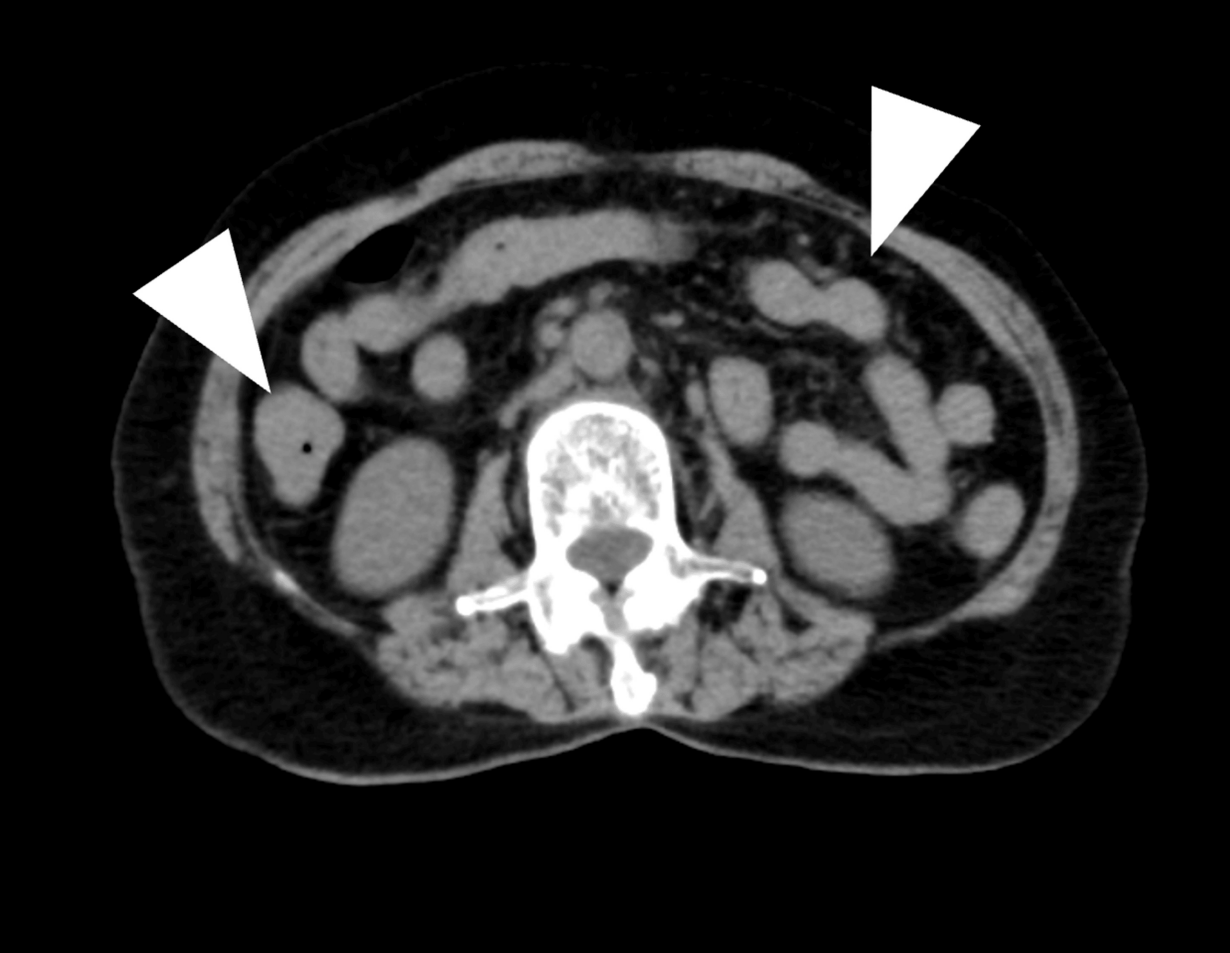
Abdominal CT in the first episode Abdominal CT performed during the first episode demonstrated subtle bowel wall edema, indicated by the white arrow, suggesting gastrointestinal involvement. CT: computed tomography

**Figure 2:**
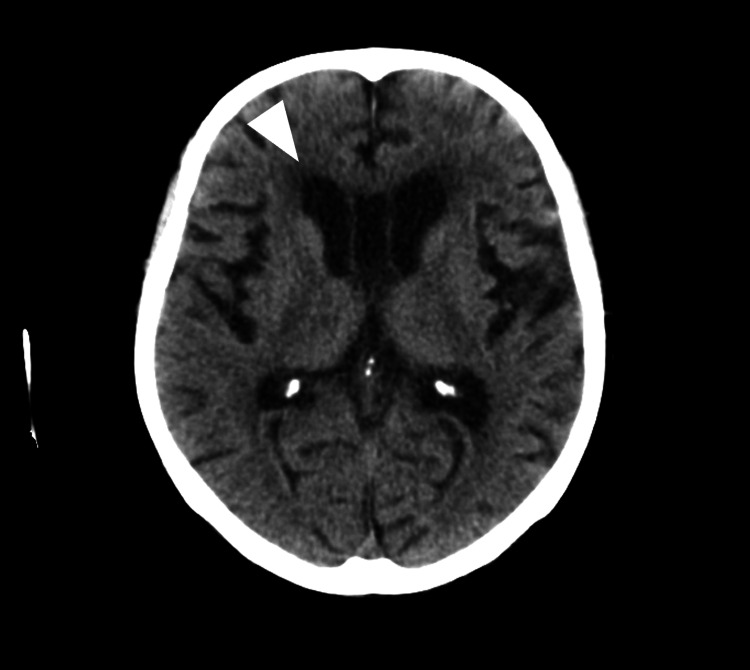
Head CT in the first episode No intracranial hemorrhage or mass effect was observed. The white arrow highlights a representative area of normal findings. CT: computed tomography

**Figure 3:**
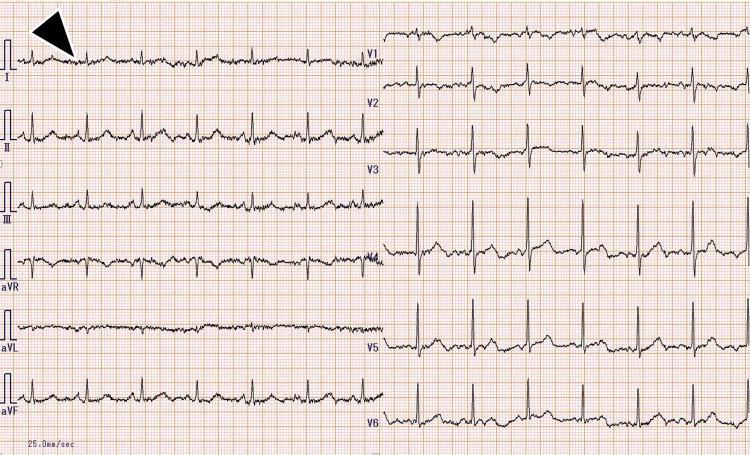
Electrocardiogram in the first episode The electrocardiogram showed normal sinus rhythm with fine baseline undulations suggestive of shivering artifact (indicated by the black arrow).

At the time of the first episode, the patient had been receiving a transdermal rivastigmine patch at a dose of 18 mg/day, prescribed by another institution. This dosage had remained unchanged for at least 32 months prior to the final episode. Although details regarding the initial titration schedule were unavailable, there had been no recent adjustments in dose or concomitant medications before the first crisis. She presented with vomiting, diarrhea, diaphoresis, and altered mental status. On arrival, vital signs were notable for hypothermia with a body temperature (BT) of 35.1°C, a blood pressure (BP) of 160/89 mmHg, a heart rate (HR) of 88 bpm, a respiratory rate (RR) of 20/min, and an SpO₂ of 100%. Physical examination revealed a soft abdomen with mild tenderness and no peritoneal signs. Laboratory studies showed the following: white blood cell (WBC) 12,000/µL, C-reactive protein (CRP) 0.22 mg/dL, electrolytes within normal limits, and serum cholinesterase (ChE) markedly reduced to 63 U/L (reference range 185-431 U/L). Abdominal computed tomography (CT) (Figure 1) revealed subtle bowel wall edema, while head CT (Figure 2) and electrocardiography (Figure 3) were unremarkable. She was diagnosed with acute gastroenteritis. Rivastigmine and all other medications were discontinued upon admission. Her gastrointestinal symptoms resolved promptly. Serial ChE monitoring showed recovery to 199 U/L by day 4. After full clinical recovery, medications, including rivastigmine, were resumed, and she was discharged.

Second episode (approximately 11 months later)

The patient was readmitted with vomiting, diarrhea, generalized weakness, drowsiness, and diaphoresis. Family members reported that she might have ingested food dropped on the ground recently treated with pesticide, raising concern for possible organophosphate poisoning. On admission, her vital signs were as follows: BP 156/68 mmHg, HR 84 bpm, BT 35.2°C, RR 12/min, and SpO₂ 96%. No miosis, hypersalivation, or tremor was observed on examination. Laboratory results showed the following: WBC 12,600/µL, CRP <0.1 mg/dL, electrolytes within normal limits, albumin 4.3 g/dL, and ChE 58 U/L (day 1). Again, rivastigmine, along with all other medications, was immediately discontinued. Symptoms improved rapidly with supportive care. Serial ChE levels rose to 138 U/L by day 2 and 303 U/L by day 3. After recovery, all medications, including rivastigmine, were resumed, and she was discharged.

Third episode (approximately 21 months after the second episode)

The patient was brought to the emergency department with impaired consciousness (Glasgow Coma Scale (GCS) E2V1M4), frequent vomiting, and profuse diaphoresis. On presentation, her vital signs were as follows: BP 163/87 mmHg, HR 78 bpm, BT 35.1°C, RR 20/min, and SpO₂ 95%. Physical examination showed no miosis but revealed chronic nasal discharge and excessive salivation, which had been ongoing for several months. Laboratory tests revealed the following: WBC 9,100/µL, CRP <0.1 mg/dL, electrolytes within normal range, albumin 3.6 g/dL, and ChE 53 U/L (day 1). Suspecting rivastigmine-induced cholinergic crisis, rivastigmine and all other medications were discontinued. The patient's vomiting and mental status improved markedly by the following day (GCS E4V4M6). ChE rose to 268 U/L by day 2 and further to 349 U/L by day 3. This time, rivastigmine was permanently discontinued, while other medications were cautiously resumed without recurrence of symptoms. Notably, chronic diarrhea and rhinorrhea also resolved following rivastigmine discontinuation. Over the next four years, she made several emergency department visits for unrelated reasons, and no recurrence of cholinergic symptoms was observed. She ultimately suffered cardiopulmonary arrest at home and was pronounced dead at the hospital.

## Discussion

In one of the largest epidemiological studies to date, Ohbe et al. analyzed a Japanese nationwide inpatient database covering approximately 40 million hospitalizations over a 69-month period [1]. They identified 235 patients with cholinergic crisis, corresponding to an incidence of approximately one case per 170,000 hospitalizations. Overall in-hospital mortality was approximately 6%, and about half of the patients required catecholamine administration, atropine, or mechanical ventilation, while the remaining half recovered without these interventions. The need for intensive support was associated with increased mortality and prolonged hospital stays in these patients. Although specific causative agents could not be identified due to database limitations, the authors noted that comorbid conditions such as voiding dysfunction and myasthenia gravis were present in some patients, suggesting that distigmine and pyridostigmine might have contributed to certain cases. Ohbe et al. also emphasized that early recognition of cholinergic crisis can be difficult, particularly in elderly patients, because of nonspecific clinical presentations [1]. They suggested that many cases might be misdiagnosed as other common acute illnesses, such as pneumonia, due to clinicians' limited prior experience with the condition. Indeed, in our patient, the first two episodes were initially misattributed to other conditions, including acute gastroenteritis and possible pesticide ingestion, before cholinergic crisis was correctly diagnosed during the third episode. Notably, in the first episode, abdominal CT revealed bowel wall edema, initially supporting the diagnosis of gastroenteritis. However, Sobajima et al. reported a case of cholinergic crisis with similar CT findings that led to initial misdiagnosis as gastroenteritis [6]. Thus, bowel edema does not exclude cholinergic crisis and must be interpreted alongside clinical and laboratory data.

In this case, serial monitoring of serum cholinesterase levels provided a critical diagnostic clue. The patient exhibited marked ChE suppression during each episode, with rapid normalization following drug discontinuation, strongly supporting the diagnosis of cholinergic crisis. However, it is important to recognize that ChE levels may not always be suppressed in all cases of ChEI-induced toxicity. Notably, previous reports have documented galantamine- or donepezil-induced cholinergic crises despite normal ChE levels, highlighting the limited utility of routine ChE assays in such cases [7,8]. This discrepancy likely reflects differences in pharmacologic selectivity, as both galantamine and donepezil predominantly inhibit acetylcholinesterase rather than butyrylcholinesterase, the latter of which is primarily measured in routine ChE assays. Thus, while ChE monitoring can serve as a valuable adjunctive tool, clinicians should maintain a high index of suspicion for cholinergic crisis even when ChE levels are not markedly reduced.

Another notable feature in this case was the presence of chronic low-grade symptoms, including persistent diarrhea and rhinorrhea, which may have indicated mild autonomic involvement preceding the overt crises. These prodromal features resolved after the permanent discontinuation of rivastigmine. Subtle autonomic symptoms of this nature should prompt clinicians to carefully review medication regimens in elderly patients, particularly when nonspecific gastrointestinal symptoms are present. In this context, the potential contribution of concomitant medications must also be considered. No known significant drug-drug interactions with rivastigmine that could explain the cholinergic crises were identified among the concomitant medications, including gastroprotective agents, antitussives, herbal medicines, and antihistamines [9]. These agents do not significantly increase rivastigmine concentrations, as rivastigmine is primarily metabolized by cholinesterase-mediated hydrolysis rather than cytochrome P450 enzymes [9,10].

In Japan, the rivastigmine patch is labeled by the total drug content (e.g., 18 mg), whereas in the United States and some other countries, it is labeled by the average drug delivery rate over 24 hours (e.g., 9.5 mg/24 h). Therefore, the 18 mg patch used in this case corresponds to the 9.5 mg/24 h dose commonly cited in international literature and clinical guidelines [11].

## Conclusions

Rivastigmine-induced cholinergic crisis is an extremely rare complication, particularly when occurring under routine therapeutic dosing of the transdermal formulation. This case highlights the diagnostic challenges associated with cholinergic crisis in elderly patients, where nonspecific gastrointestinal and autonomic symptoms may lead to misdiagnosis or delayed recognition. Although serial serum cholinesterase measurements can provide useful diagnostic clues, clinicians should be aware that cholinergic crisis may occur even in the absence of significant ChE suppression, particularly with selective acetylcholinesterase inhibitors. Clinicians should maintain vigilance for cholinergic toxicity in elderly patients treated with ChEIs, particularly when faced with unexplained gastrointestinal or autonomic symptoms, even in the absence of overt overdose.
